# Effects of different feeding frequency on Siamese fighting fish (*Betta splenden*) and Guppy (*Poecilia reticulata*) Juveniles: Data on growth performance and survival rate

**DOI:** 10.1016/j.dib.2020.106046

**Published:** 2020-07-20

**Authors:** Nor Hakim Norazmi-Lokman, Asshatul Ain Baderi, Zakirah Mohd Zabidi, Abdul Wahid Diana

**Affiliations:** a Faculty of Fisheries and Food Sciences, Universiti Malaysia Terengganu, 21030 Kuala Terengganu, Terengganu, Malaysia; b Fisheries and Aquaculture Centre, Institute for Marine and Antarctic Studies, 7053 Taroona, Tasmania, Australia; c Kuala Terengganu, 21030 Terengganu, Malaysia

**Keywords:** Ornamental fish, Aquaculture, Husbandry, Betta fish, Livebearing Fish

## Abstract

Optimal feeding frequency in aquaculture is vital for the sustainable and economical production of healthy, high-quality fish. This article described the growth performance and survival rate data in the juvenile phase of two commercially important ornamental fish species; *Betta splendens* (Siamese fighting fish) and *Poecilia reticulata* (Guppy) reared at different feeding frequency. Thirty days old juveniles of both species were randomly distributed into 12 3 L rectangular plastic tank (*n* = 10 fish/tank; three replicates per feeding frequency) where they were subjected to four different feeding frequency treatments (1 meal/day (T1), 2 meal/day (Control), 3 meal/day (T2) and four meal/day (T3)) using commercial ornamental fish micropellets for 60 days. The juvenile's weight and length were measured once a week while the number of live fish were recorded daily. The amount of feed intake was also recorded by weighing in the weight of micropellet left after feeding. At the end of the experimental period, the specific growth rate (SGR), food conversion ratio (FCR) and condition factor (K) were calculated for growth performance using the weight, length and feed intake data while the survival rate was calculated using the number of live fish data. Normality test, One-way ANOVA analysis followed by Tukey pos-hoc test were then performed on the data obtained from the calculation of SGR, FCR, K an survival rate. The data presented in this article will aid the rearing process of both species’ juveniles for commercial, experimental and personal usage purpose.

Specifications Table

**Table:** 

Subject	Aquatic Science
Specific subject area	Aquaculture
Type of data	Graph
	Table
How data were acquired	Data were acquired from laboratory experiment where physical measurements as well as calculation for growth rate, survival rate and feed conversion rate were conducted. Statistical analysis was done using IBM SPSS statistics software version 25 (IBM, US)
Data format	Raw
	Analysed
Parameters for data collection	Growth performance data: Measurement of body weight and total length of the fish
	Feed utilization: Amount of feed intake (weight) and measurement of fish weight.
	Survival rate data: Quantity of live fish
Description of data collection	Growth performance data: Measurement of body weight and total length were done once a week for eight weeks
	Feed utilization: Amount of feed consumed (weight) and measurement of fish weight were done at the end of the 60-day experimental period
	Survival rate data: Quantity of live fish were counted daily for 60 days
Data source location	Institution: Faculty of Fisheries and Food Sciences
	City/Town/Region: Kuala Terengganu, Terengganu
	Country: Malaysia
	Latitude and longitude (and GPS coordinates, if possible) for collected samples/data: Freshwater Hatchery, Faculty of Fisheries and Food Sciences, Universiti Malaysia Terengganu: 5°24′36.2″N 103°05′20.2″E
Data accessibility	1. With the article
	2. Repository name: Mendeley Data
	Data identification number: DOI: 10.17632/gbxfpzgtsk.2
	Direct URL to data: https://data.mendeley.com/datasets/gbxfpzgtsk/2

## Value of the data

• Betta and guppy are important species for commercial and experimental purpose due to their unique biology. These data will aid the rearing process of both species’ juveniles for commercial, experimental and personal usage purpose.
• These data will benefit farmers, breeders, scientist and ornamental hobbyist.
• These data can be used by scientist to ensure the optimum performance/conditions of their fish (in terms of growth and survival) during experiments thus guaranteeing the success of their research.

Having the data on the optimum feeding frequency of both species can help farmers, breeders and aquarium enthusiast to increase production and profits while reducing their production cost associating to feed.

## 1 Data description

**Fig. 1:**
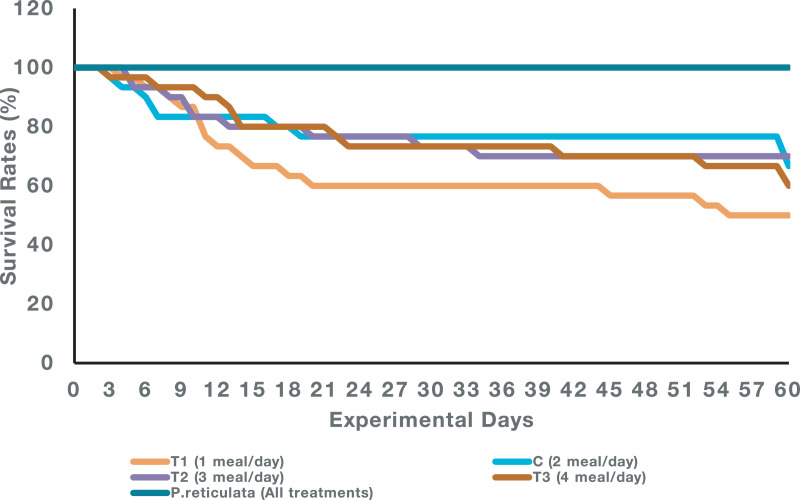
Survival rate curve of *B. splendens* and *P. reticulata* juveniles fed with different feeding frequency throughout the 60 days experimental period.

Raw data on the weight, growth, feed intake and number of fish survived throughout the 60 days experimental period are as in [1]. Table 1 describe the results on the specific growth rate (SGR) in term of weight and length, food conversion ratio (FCR), condition factor (K) and survival rate of both species at the end of the experiment which was calculated and analysed based on [1]. All data in the table is presented as mean ± standard deviation. Meanwhile, the survival rate curve of both species for 60 days is represented in Fig. 1.

**Table 1:** The SGR (weight and length), food conversion ratio, condition factor and survival rate of *B. splendens* and *P. reticulata* juveniles at different feeding frequency after 60 days of experimental period.

**Parameters**	***B. splendens***					***P.reticulata***				
	SGR Weight (mg) (% day^−1^)	SGR Length (mm) (% day^−1^)	FCR	K	Survival Rate (%)	SGR Weight (mg) (% day^−1^)	SGR Length (mm) (% day^−1^)	FCR	K	Survival Rate (%)
T1 (1 meal/day)	3.297 ± 0.263	15.723 ± 0.856^a^	2.256 ± 0.871	1.560 ± 0.166	50 ± 20.00	4.010 ± 0.086^bc^	1.370 ± 0.119^a^	7.498 ± 0.859^a^	3.827 ± 0.025^b^	100
C (2 meal/day)	3.591 ± 0.186	17.159 ± 1.823^ab^	2.222 ± 0.559	1.377 ± 0.149	66.67 ± 20.82	3.751 ± 0.078^a^	1.720 ± 0.031^b^	20.466 ± 2.154^c^	2.077 ± 0.113^a^	100
T2 (3 meal/day)	3.856 ± 0.153	20.062 ± 1.661^b^	1.521 ± 0.326	1.161 ± 0.338	70 ± 26.46	3.866 ± 0.019^ab^	1.843 ± 0.037^b^	21.443 ± 0.558^c^	2.089 ± 0.032^a^	100
T3 (4 meal/day)	3.418 ± 0.182	15.243 ± 2.404^a^	2.177 ± 0.524	2.145 ± 1.403	60 ± 10.00	4.119 ± 0.053^c^	1.869 ± 0.025^b^	15.883 ± 1.183^b^	2.168 ± 0.048^a^	100

Means within a column with different superscripts are significantly different with one another (*P* < 0.05).

## 2 Experimental design, materials and methods

### 2.1 Location of study

The study was conducted at the Ornamental Fish Breeding Unit, Faculty of Fisheries and Food Sciences freshwater hatchery, Universiti Malaysia Terengganu.

### 2.2 Fish source and maintenance

The ornamental fish breeding unit maintained both species fish stocks which was obtained from routine breeding. 20–30 individuals of each species healthy broodstock were randomly chosen for breeding purposes.

#### 2.2.1 Betta splendens

10 pairs of healthy mature *B. splendens* broodstock (4 to 6 cm in total length) were paired randomly in rectangular plastic tanks filled with 3 L of freshwater in a static indoor water system. Naturally dried brown Catappa/India Almond leaves (*Terminalia cattapa*) were included in each aquarium (1 leaf per aquarium) to lower the water pH thus inducing natural breeding. Water quality was monitored daily (API Master Freshwater testing kit, US) and maintained at 0 ppt, pH 5–6, 26–28 °C. Water changes were made when necessary to avoid stress and disturbance to the fish breeding process. Broodstocks were fed with tropical fish micropellet (Sanyu Ichiban, China) till satiation twice daily at 9 am and 4 pm. Once every two days, dried blood worms were fed as a supplement to the broodstock.

The female was first separated from the male in the same tank by using clear plastic cups. Once both sexes showed interest to breed (male preparing bubble nest and swimming close to females), the female were release from the plastic cup for breeding to occur which usually take place immediately or up to three days depending on fish. Male fish usually guarded and care the eggs until they hatched (24–36 h). Once the larvae hatched and started swimming freely, they were carefully transferred into a 5 L stock tank where they were pooled with other larvae of the same age at the stocking density of 5 fish/L. The same water quality parameters used in broodstock propagation applies.

The larvae were fed with freshly prepared artemia (Bio-Marine, USA) twice a day (same time as the broodstock) ad libitum. Weaning into commercial micropellets (Sanyu Ichiban, China) takes place at when the juveniles are at 3 weeks old. Once they reach 30 days old, 120 juveniles were chosen randomly for the trial.

#### 2.2.2 Poecilia reticulata

Since female *P. reticulata* has the ability to store male sperm, only female broodstock (4–5 cm total length) were chosen and acclimatized for two weeks prior to parturition. The females were chosen based on the appearance of its gravid spot [2]. They were reared in a 5 L rectangle plastic tanks filled with freshwater (0 ppt, pH 6.5–7, 26–28 °C) at the stocking density of 2 ind/L in a stagnant indoor water system. Gentle aeration was provided in each tank. Water quality was monitored daily (API Master Freshwater testing kit, US) and water changes were conducted once every three days. The broodstocks were fed till satiation twice daily (9 am and 4 pm) with commercial tropical fish micropellet (Sanyu Ichiban, China). Dried bloodworm was also offered once every two days ad libitum as a supplement. Uneaten food and faeces were siphoned out after each meal to maintain water quality. The photoperiod was set at 12 L: 12 D (light: dark).
After acclimatization, each female was housed individually in a breeding trap (green plastic net; mesh size 6 mm) fitted in a 3 L rectangular plastic aquarium filled with 2.5 L of freshwater. This were done to avoid the broodstock from eating new-born fish. The tank was monitored every morning (10 am) and evening (5 pm) for the presence of new-born juveniles since routine observation in our lab showed that parturition occurs regularly at either period.
Once parturition occurs, the female broodstock will be removed from the tanks. The new-born will be reared for three days in the same tank before being transferred into a 5 L rectangular plastic stock tanks where they will be pooled with progenies of the same age from other broodstock at the stocking density of 4 fish/L. The new-born juveniles were fed ad libitum with artemia (Bio-Marine, USA) (9 am and 4 pm) prepared fresh daily before they were gradually weaned to commercial tropical fish micropellets (Sanyu Ichiban, China) starting at 12 days after parturition (DAP). Hundred and twenty juveniles aged 30 DAP were then chosen randomly for the different frequency feeding trails. Water quality and routine water maintenance throughout the propagation of juveniles were done similar to those of broodstock.

Experimental design

**Fig. 2:**
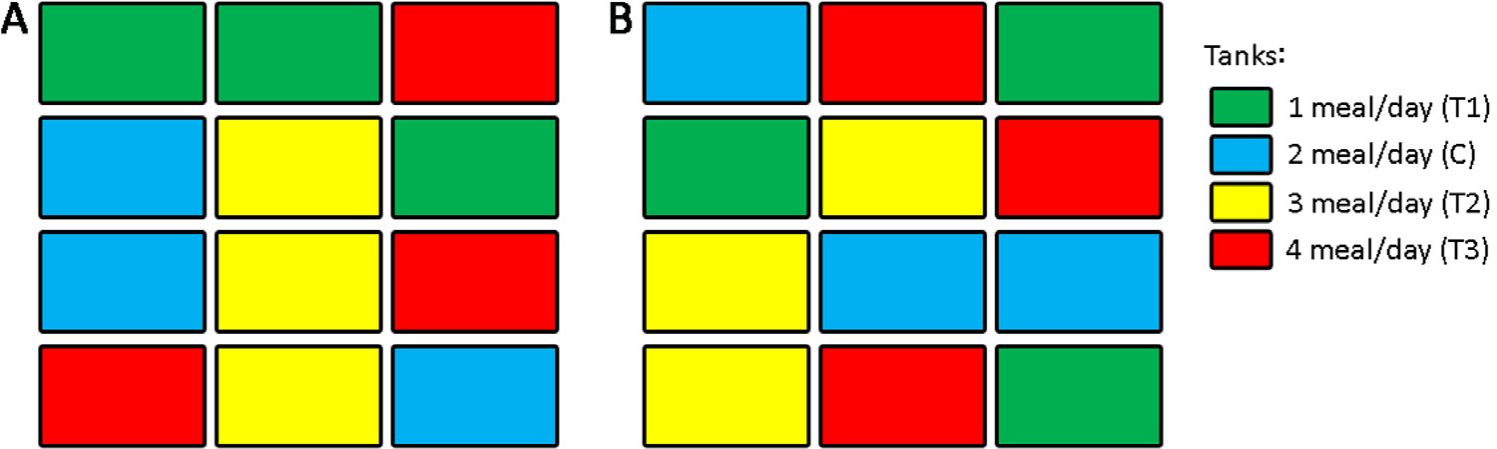
Randomized treatment tanks arrangement; A: *B. splendens* and B: *P. reticulata*.

One hundred and twenty individuals of each species were chosen randomly and equally distributed into 12 3 L rectangular plastic tank (*n* = 10 fish per tank; stoking density: *B. splendens* = 5 fish/L, *P. reticulata* = 4 fish/L). Arrangements of tanks were done based on A 3 × 4 randomized design (Fig. 2). The feeding schedule and frequency is as described in Table 2. The feeding trials of both species were conducted simultaneously for eight weeks.

**Table 2:** Feeding frequency and schedule.

### 2.3 Data collection and calculation

Measurement of body weight (gram) and total length (cm) were done once a week for eight weeks using electronic balance and a ruler while the survival was measured by counting number of live fish daily. The following formulas was used to calculate the growth performance and survival rate [3,4,5]:

$$SGR\;\left(weight\right),\;\%=\frac{log\;\left(Final\;body\;weight-Initial\;body\;weight\right)}{Experimental\;period}\times 100$$

$$SGR\;\left(length\right),\;\%=\frac{log\;\left(Final\;total\;length-Initial\;total\;length\right)}{Experimental\;period}\times 100$$

$$FCR=\frac{Total\;feed\;consumed}{Weight\;gain}$$

$$Condition\;Factor,\;K=\frac{Fish\;weight}{{\left(Fish\;length\right)}^{3}}\times 100$$

$$SR,\;\%=\frac{Final\;number\;of\;fish}{Initial\;number\;of\;fish}\times 100$$

### 2.4 Statistical analysis

IBM SPSS Statistical analysis software (version 25) was used to analyse all the data. Shapiro-Wilk normality test were first conducted prior to One-way ANOVA analysis followed by Tukey post-hoc test (where applicable) to determine the differences in the growth performance and survival rate of the different feeding frequency [6]. Differences were considered to be significant at *P* < 0.05. All analysed data are presented as mean ± standard deviation unless otherwise stated.

## 3 Ethics statement

The authors confirm that all experiments comply with the ARRIVE guidelines and were carried out in accordance with the U.K. Animals (Scientific Procedures) Act, 1986 and associated guidelines, EU Directive 2010/63/EU for animal experiments, or the National Institutes of Health guide for the care and use of Laboratory animals (NIH Publications No. 8023, revised 1978)].

Moral and ethical aspect of the research such as animal handling and minimum amount of fish needed for valid statistical analysis also complied with the Research Ethics Guidelines of Universiti Malaysia Terengganu.

## 4 Declaration of competing interest

The authors declare that they have no known competing financial interests or personal relationships which have, or could be perceived to have, influenced the work reported in this article.

## CRediT author statement

Norazmi-Lokman: Supervision, Conceptualization, Methodology, Validation, Resources, Writing – Original Draft Asshatul Ain: Conceptualization, Methodology, Investigation, Formal analysis Zakirah: Conceptualization, Methodology, Investigation, Formal analysis Diana: Validation, Writing – Review&Editing

## Declaration of Competing Interest

The authors declare that they have no known competing financial interests or personal relationships which have, or could be perceived to have, influenced the work reported in this article.

## Appendix Supplementary materials

Image, application 1
